# Anti-Inflammatory Properties of Resveratrol

**DOI:** 10.3390/ijms262311710

**Published:** 2025-12-03

**Authors:** Mateusz Wątroba, Dariusz Szukiewicz

**Affiliations:** Department of Biophysics, Physiology & Pathophysiology, Faculty of Health Sciences, Medical University of Warsaw, 02-004 Warsaw, Poland; mateusz.watroba@wum.edu.pl

**Keywords:** resveratrol, anti-inflammatory effects, oxidative stress, inflammatory response, anti-inflammatory treatment

## Abstract

Resveratrol (RSV), a naturally occurring phytoalexin polyphenolic compound, continues to attract the attention of researchers due to its therapeutic potential, including anti-inflammatory effects. The focus of this work is to critically review RSV’s anti-inflammatory effects at cellular and molecular levels and its clinical and physiological implications as elucidated by in vivo and in vitro studies to identify some unresolved issues. Reference was made to the dietary recommendations of RSV, its poor bioavailability, and high metabolism, limiting the therapeutic use of RSV and requiring formulation strategies for improving its clinical usefulness. Issues related to the interpretation of the results of studies on the anti-inflammatory activity of RSV were also discussed. The cellular response to RSV and the probable molecular pathways associated with it were analyzed separately. Another point for further investigation is the fact that not all the effects found in vitro or in animal models are replicated in clinical studies.

## 1. Introduction

The search for bioactive compounds with therapeutic potential continues to be an active area of research [1]. Resveratrol (RSV) is a phytoalexin polyphenolic compound commonly found in grapes, berries, and peanuts. Its activities in several areas of health and disease, including its anti-inflammatory properties, make it a compound of interest [2,3]. The basis for this review is the concept of how RSV exerts its anti-inflammatory effects in mammals at cellular and molecular levels.

Inflammation is the body’s natural response to infection, injury, or cellular stress. However, unregulated inflammation is involved in the pathogenesis of several human disorders, including cardiovascular, metabolic, autoimmune, and neurodegenerative diseases [4,5]. Anti-inflammatory strategies can be used for the treatment of various medical conditions. Research demonstrates that RSV decreases production of pro-inflammatory cytokines and influences inflammatory signaling pathways, inhibiting cyclooxygenase enzymes, downregulating nuclear factor kappa B (NF-κB) activity, and regulating toll-like receptor signaling, suggesting the practical application of this drug in several clinical and physiological cases [3].

Previous studies suggest that RSV may be beneficial for several health conditions, possessing effects of being antioxidant, cardioprotective, neuroprotective, and now it has been linked to anti-inflammatory effects by inhibiting pro-inflammatory cytokines like tumor necrosis factor alpha (TNF-α), interleukins (IL-6, IL-1β), and inducible nitric oxide synthase (iNOS), and increasing anti-inflammatory cytokines such as interleukin-10 (IL-10), as well as targeting molecular pathways involved in inflammation such as nuclear factor kappa-light-chain-enhancer of activated B cells (NF-κB) and AMP-activated protein kinase/sirtuin 1 (AMPK/SIRT1) pathways [6,7,8,9]. However, the poor bioavailability and high metabolism of RSV limit its use, thereby requiring formulation strategies for improving its clinical use [10].

The limitation of this work to presenting the mechanisms of RSV’s anti-inflammatory action is deliberate. The increasingly well-known anti-inflammatory effects of RSV are essentially the basis for its other beneficial biological effects, secondary to suppressing the inflammatory response. Our approach proves to be innovative, complementing other review papers organizing the latest knowledge on RSV, which describe all RSV actions without referring to their cause-and-effect relationship with inflammation.

The focus of this work is to critically review RSV’s anti-inflammatory effects at cellular and molecular levels and its clinical and physiological implications as elucidated by in vivo and in vitro studies to identify some unresolved issues.

To review the literature, a broad strategy was taken to search databases of published, peer-reviewed experimental and observational research studies assessing the potential benefits of RSV on the prevention of inflammation. Literature sources include the use of observational and experimental interventions, as well as molecular mechanistic studies. The searches were based on cytokine profiling, gene expression, enzymatic activities, and/or inflammatory cellular responses. The methodology of comparative literature evaluation was applied to extract and compare convergent and divergent observations.

## 2. A Brief Introductory Overview of Inflammation and Anti-Inflammatory Treatment

### 2.1. Inflammation

Inflammation is a natural protective reaction of the organism in response to infections or injuries; it basically helps maintain tissue homeostasis in stressful conditions [11,12]. This complex, strictly regulated process serves as a quick protective mechanism to prevent possible pathogens, limit tissue damage, as well as stimulate reparatory processes; therefore, inflammation is of utmost significance for human health [13]. Although details of inflammatory response depend on the nature of the initial insult and its location within organism, all of them share common steps, such as ❶ pattern recognition by pattern-recognizing receptors (PRRs), ❷ activation of metabolic pathways inducing inflammatory response, ❸ release of inflammation markers, ❹ recruitment of cells taking part in the inflammation, and ❺ the inflammation occurring in the affected tissues, which is often accompanied by the impairment of their functions [14,15]. This complex sequence of events results in main symptoms of inflammatory response: pain, active hyperemia, edema, and loss of function of the affected organ [16].

Inflammation may be acute or chronic [11]. Acute phase is usually initiated by cells already located in tissues, detecting pathogens or injuries, and subsequently sending chemical signals which enhance local response and recruit other cells [13]. Molecular and cellular processes occurring during acute inflammatory response are usually effective in the restoration of tissue homeostasis and thus leading to resolution of the inflammatory response [17]. However, an alteration or prolongation of inflammatory response may result in chronic inflammation, which can sometimes do more harm to the host organism than the pathogen itself, even if it is not very intensive [18]. Low-grade inflammation may last for a lifetime because of chronic or recurrent infections, and emerging evidence suggests that such chronic inflammation can play an essential role in the pathogenesis of many chronic diseases, including obesity and metabolic syndrome, cardiovascular diseases, and neurodegeneration [19,20,21,22,23]. In addition, research studies indicate some correlations between chronic inflammation and some kinds of cancer [13,24].

### 2.2. Typical Anti-Inflammatory Treatments

#### 2.2.1. Non-Steroid Anti-Inflammatory Drugs (NSAIDs)

Anti-inflammatory treatment based on NSAIDs may, in many cases, be characterized by the occurrence of side effects and limited effectiveness [25,26,27]. Those drugs are strong inhibitors of cyclooxygenases 1 and 2 (COX-1 and COX-2). While COX-2 is indeed induced by inflammatory response to initiate biosynthesis of pro-inflammatory prostaglandins (PGE2 and PGD2), COX-1 is a constitutive isoform of the enzyme, involved in homeostasis maintenance processes. Since NSAIDs inhibit both isoforms of the enzyme, their prolonged use may lead to the damage of gastrointestinal tract lining, which is their main side effect [28]. This is why selective COX-2 inhibitors have been introduced. Those selective inhibitors, called coxibs, have reduced gastrointestinal tract side effects, but are potentially more cardiotoxic and hepatotoxic [26,29]. Because of this, some medications already approved by the Food and Drug Administration (FDA) have been retracted from sale [30,31].

#### 2.2.2. Glucocorticoids

In addition to NSAIDs, glucocorticosteroids comprise another standard therapy alleviating inflammatory response. Glucocorticoids reduce inflammation by inhibiting the production of pro-inflammatory molecules and boosting anti-inflammatory mediators through several mechanisms [32,33]. They bind to the glucocorticoid receptor (GR), which then moves to the nucleus to act as a transcription factor, either by directly blocking genes that promote inflammation or by upregulating genes that suppress it [34,35]. These drugs also prevent the initial inflammatory response, reduce the migration of immune cells to affected sites, and promote the resolution of inflammation [36,37]. Moreover, recent studies also indicate a non-nuclear role of GR in anti-inflammatory action. It has been shown that the loss of a cytosolic protein–protein interaction with GR allows for glucocorticoid-mediated anti-inflammatory activity rather than the presence of GR in the nucleus [37,38].

However, resistance to glucocorticoid anti-inflammatory action is a significant hindrance in the effective treatment of many diseases. Furthermore, glucocorticoid side effects may include metabolic syndrome, osteoporosis, and dysfunction of musculoskeletal system, gastrointestinal system, cardiovascular system, neuropsychiatric system, and immune system [39,40].

This is why there is an urgent need to find new, safe, and effective anti-inflammatory substances [11]. This review is devoted to the naturally occurring polyphenol, resveratrol (RSV), whose use as a phytopharmaceutical in the treatment of inflammation is the subject of numerous studies [41,42,43].

## 3. Overall Characteristics of RSV

**Figure 1:**
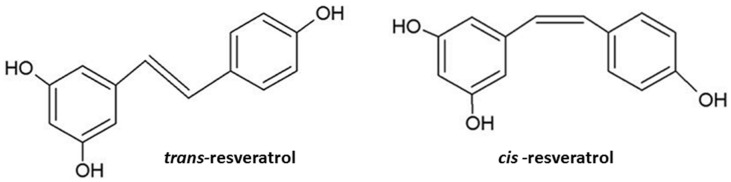
Chemical structure of the resveratrol (RSV) molecule including isomeric forms *trans*- and *cis*-.

RSV, a naturally occurring phytoalexin polyphenolic compound, is found mostly in grapes, mulberries, peanuts, rhubarb, and in many different kinds of berries. The biologically active trans-isomer is the most abundant and efficacious, and has a greater physiological effect than the cis-isomer [44] (Figure 1).

This preference towards the trans-isomer for therapeutic research is clearly reflected in most of the related published reports. Due to its antioxidant, anti-inflammatory, cardioprotective, neuroprotective, and cancer-preventive properties, RSV is of particular interest [44,45,46]. Hence, the importance of further investigating the differences in the activities of trans- and cis-resveratrol in relation to the context of their therapeutic use must be emphasized [12,45].

### 3.1. Mechanisms of RSV’s Anti-Inflammatory Actions

RSV’s anti-inflammatory effects stem from cellular mechanisms and intricate molecular pathways that influence immune activity, protect tissues, and manage systemic inflammation. Exploring these effects illuminates how RSV impacts immune signaling, modulates gene expression, and influences signaling cascades, thereby clarifying its potential in the therapeutic setting. The intricacies of these effects contribute to an understanding of RSV’s broader biological actions and how it interacts within the immune system [46,47].

#### 3.1.1. Cellular Response

The cellular response to RSV’s anti-inflammatory effect displays a multifaceted interaction between immune cell modulation, barrier tissue protection, and systemic anti-inflammatory effects [48,49]. An important feature of RSV’s anti-inflammatory effects lies in its ability to modulate immune cell cytokine secretion and phenotype. RSV diminishes pro-inflammatory IL-1β, IL-6, TNF-α, INF-γ cytokines and promotes the expression of anti-inflammatory IL-10 and IL-4. The downregulation of IL-6, pro-inflammatory chemokines CCL3, and INF-γ has been observed in PBMCs, following long-term supplementation with RSV [50]. The enhancement of IL-10 and the decline in INF-γ, IL-22, and IL-17A have been reported in the irradiated rat liver model [51]. In vitro studies revealed that RSV decreases inflammatory cytokine production in lipopolysaccharide (LPS)-stimulated RAW264.7 macrophages and mast cells stimulated by IL-33 [52,53]. However, these beneficial effects were not observed for all cytokines or cellular types at all tested doses [52,53]. The cytotoxic effect of RSV was observed on RBL-2H3 mast cells and RAW264.7 macrophages at high concentrations [52,53]. An increment in IL-10 mRNA in Kupffer cells following the administration of RSV in rats has been reported. All these results emphasize the immunomodulatory potential of RSV in reference to inflammation [51].

The prevention of inflammatory damage of barrier and neural tissues in response to RSV treatment has been reported due to its effect on controlling leukocyte adhesion and cytokine secretion. In the rodent retina model, pre-treatment with RSV reduces the adhesion of leukocytes to retinal vessels and decreases levels of MCP-1 and ICAM-1, which indicate reduced inflammatory processes [54]. In an in vitro blood–brain barrier model, the treatment with RSV decreased the release of astrocytic inflammatory cytokines, IL-1α, IL-1β, IL-2, IL-4, IL-6, and IL-8, at various glucose concentrations, suggesting its protective effect during the neuro-inflammatory insult caused by metabolic imbalances [42]. Maternal immune activation mouse model has demonstrated that RSV treatment decreases LPS-stimulated elevation in inflammatory markers such as IL-1β, IL-6, and TNF-α in the hippocampus and recovers the expression of synaptic and neurotrophic markers [55]. Therefore, pre-treatment with RSV could lead to an attenuation of inflammation and damage in retinal and neural tissues.

In addition to cellular and molecular responses, the reduction in pro-inflammatory cytokines and enhancement in the number of endothelial cells were observed by RSV administration, following irradiation [51]. RSV enhances the anti-inflammatory activity by decreasing macrophage and monocyte production of pro-inflammatory cytokines in multiple neurodegenerative diseases, for example, Alzheimer’s disease, and enhances the production of anti-inflammatory mediators, such as IL-4 and MDC in the CSF [56]. It inhibits the TLR4/MyD88 pathway, leading to the macrophage M1 to M2 polarization [52].

Several clinical trials confirmed the effect of RSV- and RSV-containing products, such as grape extracts, in improving inflammatory processes in multiple diseases such as cardiovascular and metabolic diseases. Clinical trials showed that long-term administration of grape extracts with RSV inhibits the expression of pro-inflammatory cytokines and microRNAs, decreases several inflammatory markers, and promotes leukocyte immunomodulation [50]. It has been shown that RSV can decrease vascular inflammation, decrease platelet activation, and improve vasoconstriction and vasodilation, which are effects that can aid in atherosclerosis prevention [47]. RSV has been shown to inhibit the expression of adhesion molecules, such as ICAM-1, and the release of the pro-inflammatory chemokine MCP-1 in in vivo models [54].

One of the causes of inflammation is the imbalance in oxidative stress and antioxidant capacity. In a clinical trial study with type 2 diabetes patients, RSV administration lowers oxidative stress marker levels such as lipid peroxides and malondialdehyde and promotes antioxidant production, increasing glutathione peroxidase and catalase activity [57]. Similar anti-inflammatory and antioxidative stress effects of RSV have also been noted in metabolic syndrome patients [47]. Several studies have pointed out that RSV exerts antioxidant activity in the body. RSV in the human study caused reduced levels of malondialdehyde in plasma of patients with diabetes, a hallmark of reduction in oxidative stress [47]. Thus, RSV could be used to ameliorate diabetes or other metabolic diseases in a way that would not be seen with some other antioxidant treatments that do not have a robust anti-inflammatory response.

Lastly, clinical trial and in vitro data have indicated that RSV not only has anti-inflammatory but also immunomodulatory effect in both autoimmune and viral infections. RSV has shown efficacy in decreasing pro-inflammatory cytokines in addition to SARS-CoV-2 replication, in vitro, through the increased activation of NK and cytotoxic T lymphocytes, therefore decreasing the levels of pro-inflammatory cytokines through NF-κB modulation and by increasing cytotoxic activity of immune cells [47,58]. RSV also reduces inflammatory and lesion sizes and diminishes autoimmune responses by preventing cell-mediated immune-related damage in multiple autoimmune diseases such as rheumatoid arthritis and type 1 diabetes [59]. Therefore, RSV has shown a great anti-inflammatory and immunomodulatory effect by modulating immune cell function to ameliorate the inflammatory responses in many infectious and autoimmune-related diseases. Although these benefits were reported by several studies, the precise mechanisms of action for RSV, as well as optimal administration routes and concentrations in response to certain infections and inflammations, must be further explored in in vitro and in vivo studies.

In conclusion, the cellular response to RSV’s anti-inflammatory effect has shown its potential as an immunomodulator through the regulation of inflammatory pathways, prevention of inflammatory damage in retinal and neuronal cells, and modulation of immune cell phenotype and activity to ameliorate inflammation across a multitude of chronic and acute diseases.

#### 3.1.2. Molecular Pathways

**Figure 2:**
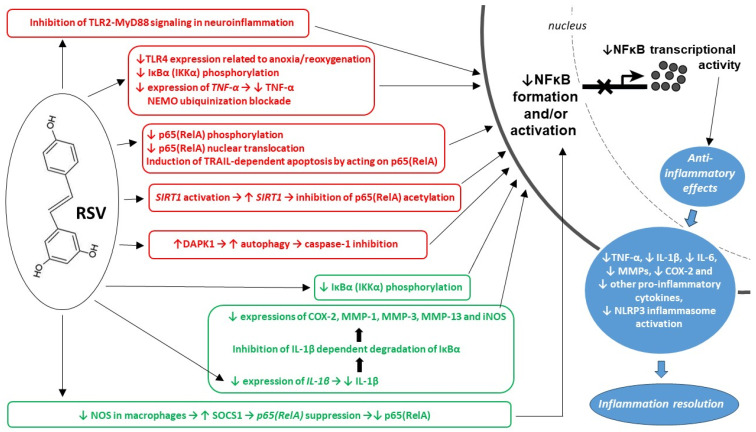
Anti-inflammatory effects of resveratrol (RSV) related to its influence on the NF-κB-dependent signaling pathways. The canonical NF-κB activation pathway is marked in red, while the green color indicates signaling through both signaling pathways (canonical and alternative) and/or another pathway(s). The effects of inhibition of NF-kB transcription (marked with a crossed x) are summarized in boxes with a blue background. COX-2—cyclooxygenase 1; DAPK1—death-associated protein kinase 1; IκBα (IKKα)—inhibitory of nuclear factor kappa B kinase alpha; IL-1β, IL-6—interleukins 1β and 6, respectively; iNOS—inducible nitric oxide synthase; MMP-1, MMP-3, MMP-13—matrix metalloproteinases 1, 3 and 13; MMPs—matrix metalloproteinases; MyD88—myeloid differentiation primary response 88, an adaptor protein; NEMO—nuclear factor kappa-light-chain-enhancer of activated B cells (NF-κB) essential modulator; NFκB—nuclear factor kappa-light-chain-enhancer of activated B cells; NLRP3 inflammasome—nucleotide-binding oligomerization domain (NOD), leucine-rich repeat (LRR)-containing protein (NLR) family member 3 inflammasome; NOS—nitric oxide synthase; p65(RelA)—transcription factor p65, also known as nuclear factor NF-κB p65 subunit (RelA proto-oncogene); SIRT1—sirtuin 1; SOCS1– suppressor of cytokine signaling 1; TLR2, TLR4—toll-like receptor 2 and 4, respectively; TNF-α—tumor necrosis factor alpha; TRAIL—tumor necrosis factor-related apoptosis-inducing ligand. Up and down arrows are typical indications of increase or decrease, respectively.

The role of RSV in modulating molecular pathways involved in the inflammatory response has been extensively researched [45,60,61,62,63,64]. One of the major mechanisms is its inhibition of NF-κB signaling in pro-inflammatory gene expression (Figure 2). NF-κB mediates the induction of various pro-inflammatory genes, so the downregulation of this signaling pathway through the downregulation of NF-κB, IKK-α, and IKB-α levels results in reduced expression of TNF-α and IL-6 and therefore lessens chronic inflammation [65].

Some studies on RSV mechanisms of action towards NF-κB have discovered that RSV may also act on NF-κB-related kinases [66,67,68]. Death-associated protein kinase 1 (DAPK1), as a calcium-calmodulin-dependent serin-threonine kinase, has many functions and takes part in various physiologic and pathologic processes, such as cell necrosis, apoptosis, autophagy, and innate immunity [69,70]. DAPK1 has been found to inhibit NF-κB activation and pro-inflammatory cytokine expression induced by TNF-α and LPS [71,72]. Turning off DAPK1 with siRNA blocks RSV-induced autophagy, without affecting the extent of phosphorylation of AMPK as another target molecule for RSV. In human dermal fibroblasts, RSV-induced autophagy can be dependent on DAPK1 indeed, which gives rise to the hypothesis that anti-inflammatory properties of RSV may also depend on its effect on DAPK1 [73].

Inflammatory stress induced by high-glucose levels, a contributing factor to chronic low-grade inflammation in diabetic patients, can also be attenuated with RSV via the reduction in NF-κB [57]. This observation links metabolic regulation to anti-inflammatory activity, supporting its potential as a supplement to address dual challenges of insulin resistance and inflammation.

Experimental data also suggest that RSV mediates inhibition of inflammatory transcription factors, decreases phosphoenolpyruvate carboxykinase (PEPCK), and increases glucokinase (GCK) expression to modulate inflammatory signaling [68].

RSV has shown to selectively downregulate TNF-α and IL-6 expression at the mRNA and protein level [68], which may be valuable in inflammatory diseases in which the main contributors to pathology are TNF-α and IL-6. Moreover, RSV downregulates endothelial and immune cell activation markers to reduce leukocyte recruitment and infiltration into tissues [68], further supporting its activity against vasculitis.

RSV regulates inflammatory mediators such as NO, prostaglandin E2 (PGE2), iNOS, and COX-2 by the mechanistic targeting of rapamycin (mTOR) and extracellular signal-regulated kinases ½ (ERK1/2) phosphorylation reduction in microglial and macrophage models, causing anti-inflammatory outcomes [74]. The fact that rapamycin, a highly selective inhibitor for mTOR, abolished the anti-inflammatory actions of RSV indicates that RSV might trigger some anti-inflammatory effects also by inhibition of mTOR signaling [74]. The involvement of the mTOR pathway in the anti-inflammatory effects of RSV identifies it as a potential target that warrants more exploration in other disease contexts in which inflammation is a central pathological feature, given that mTOR signaling also plays an important role in tumor development and cellular metabolism.

**Figure 3:**
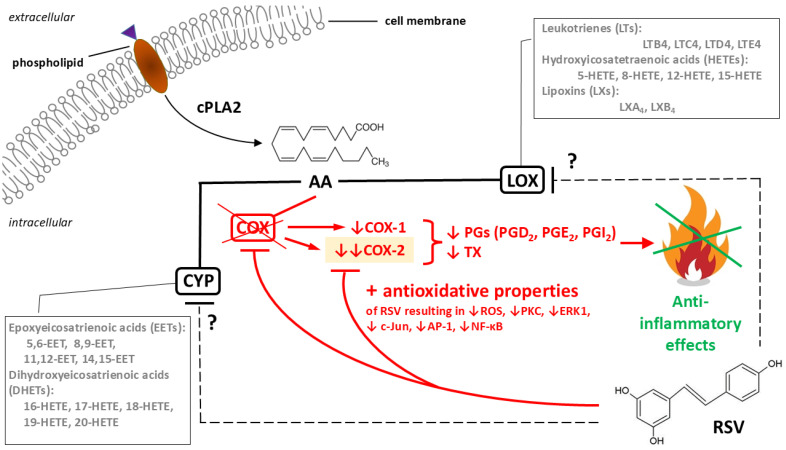
Anti-inflammatory effects of resveratrol (RSV) as a consequence of its influence on the metabolism of arachidonic acid (AA). The cleavage of cell membrane phospholipid by calcium-dependent cytosolic phospholipase A2 (cPLA2) AA is released, which is an essential fatty acid and a precursor of eicosanoids: prostaglandins (PGs), thromboxanes (TX), leukotrienes (LTs), and lipoxins (LXs). Of the three main metabolic pathways of AA, involving lipoxygenase (LOX), cytochrome P450 (CYP), and cyclooxygenases (COX), the latter has been sufficiently studied in relation to the anti-inflammatory effects of resveratrol (marked in red with a crossed X symbolizing COX inhibition). Unlike COX, the ability of resveratrol to directly inhibit LOX and CYP has not been clearly confirmed (marked with a question mark in the figure). Resveratrol is an inhibitor of both COX-1 and COX-2 but has a stronger inhibitory effect on COX-2, because the antioxidative properties of resveratrol additionally and selectively cause COX-2 to silence the transcription of the COX-2-encoding gene. Ultimately, the inhibition of COX activity by resveratrol leads to a reduction in the concentrations of pro-inflammatory mediators, mainly PGs and TX. This final anti-inflammatory effect is marked symbolically with a green crossed X as extinguishing the flame. Other abbreviations: AP-1—activator-protein-1; c-Jun—a component of the transcription factor AP-1; ERK1—extracellular signal-regulated kinase 1; NF-κB—nuclear factor kappa-light-chain-enhancer of activated B cells; PKC—protein kinase C; ROS—reactive oxygen species. Down arrows are typical indications of decrease, double down arrow indicates a stronger decrease.

RSV may inhibit COX-1, COX-2, and arachidonic acid metabolism. Inhibition of arachidonic acid metabolism plays a significant role in the anti-inflammatory effects of polyphenols, such as RSV [75,76]. This effect is primarily due to the inhibition of the COX pathway, which largely limits the production of active arachidonic acid metabolites, such as prostaglandins (PGD2, PGE2, PGI2) and thromboxanes (TX) A2 [77]. There is some evidence that both isoforms of COX (i.e., COX-1 and COX-2) are essential sources of prostaglandins [78,79]. Prostanoids produced by COX-1 account for renal homeostasis, cytoprotection, immunomodulation, and platelet function [80], while those produced by COX-2 take part in the inflammatory response [81]. It has been observed that RSV exerts its anti-inflammatory effects through inhibition of pro-inflammatory functions of cyclooxygenases and their hydroperoxidase activity [82]. RSV may inhibit cyclooxygenase and hydroperoxidase activity of COX-1 (Figure 3).

Furthermore, RSV does not affect both COX isoforms in the same way. It is a strong inhibitor of COX-1 catalytic function, but a weaker inhibitor of COX-2 peroxidase activity [83]. RSV may blunt the transcription activity induced by COX-2 promoters, including NF-κB and AP-1, as well as by protein kinase C (PKC) activation [84,85]. It has also been found that RSV may reduce oxidative stress, as well as COX-2 expression and activity through the inhibition of signaling pathways related to the activation of NF-κB, AP-1 and Janus kinase-signal transduction and transcription activation (JAK/STAT), induced in mouse skin by 12-O-tetradecanoylphorbol-13-acetate (TPA) [86]. It is known that TPA/phorbol-12-myristate-13-acetate (PMA) induce COX-2 expression through transcriptional activation of NF-κB and AP-1 [86]. The anti-inflammatory effect of RSV is related to AMPK activation, as well as the inhibition of NF-κB and COX-2-related signaling pathways in LPS-stimulated macrophages [87].

RSV can suppress ERK1/2 phosphorylation in addition to the inhibitory effects on the mTOR pathway, thus integrating an additional step in the MAPK pathway [74]. Mitogen-activated protein kinases are activated by their translocation to cell nucleus, where they phosphorylate their specific target transcription factors, such as nuclear factor erythroid 2 (NF-E2)-related factor 2 (Nrf2), NF-κB and AP-1 [88,89]. MAPK-dependent signal transduction pathways play an essential part in various biological processes, including cell proliferation and differentiation, apoptosis, inflammation, and cellular stress response [90]. MAPK constitute a family of stress-induced kinases, including enzymes such as c-Jun N-terminal kinase (JNK), ERK, big mitogen-activated protein kinase 1 [BMK1, also known as extracellular signal-regulated kinase 5 (ERK5) or mitogen-activated protein kinase 7 (MAPK7)], and p38 protein kinase [91,92]. Among them, p38 protein kinase is activated by several pro-inflammatory stimuli, such as oxidative stress, ultraviolet B radiation (UVB) and pro-inflammatory cytokines [93,94]. RSV inhibitory effect probably includes the inhibition of the MAPK-dependent cytosolic cascade of phospholipase A2-arachidonic acid (AA)-TxA2-[Ca^2+^], as well as NO/cGMP signaling pathways, which results in the inhibition of phospholipase C and PKC [95]. RSV acting through the MAPK pathway showed a protective effect on retinal ganglion cells by preventing apoptosis induced in vitro by hydrogen peroxide [96]. It has also been observed that RSV may reduce ROS accumulation, inflammation, and angiogenesis both in vivo and in vitro, thus showing some preventive properties against rheumatoid arthritis [97]. In addition, RSV can alleviate inflammation within the central nervous system (CNS), induced by injury or damage, through inhibition of pro-inflammatory mediators released from glial cells, as well as p38 MAPK activation [98,99].

RSV suppresses inflammatory signaling and downstream mediators by multiple mechanisms of action and at different regulatory levels. Thus, whether the potential for adverse effects can be avoided must be clarified for clinical translation of this approach.

AP-1 is another transcription factor which usually consists of one Jun family molecule (c-Jun, JunB, or JunD) and one Fos family molecule (c-Fos, FosB, Fra1, or Fra2) [100]. AP-1 regulates several processes, including cell proliferation, differentiation, apoptosis, and inflammation [101]. AP-1 may be activated by various extracellular stimuli, while RSV can block PMA or TNF-α-induced activation of AP-1-dependent gene expression [102,103]. RSV can inhibit PMA-induced IL-8 production, both at the level of mRNA and protein biosynthesis, which suggests that inhibition of IL-8 gene transcription by RSV results in part from inhibition of AP-1 activation [104]. Reducing the activity of NF-κB correlates with AP-1 inhibition, whereas RSV anti-inflammatory and anti-cancer properties may be in part related to blocking the activation of NF-κB, AP-1, and their related kinases [102]. Moreover, it has been found that inhibitory activity of RSV is greater than that of dexamethasone, because of the inhibition of the transcription of NF-κB and AP-1-dependent proteins, as well as the cAMP response element-binding protein (CREB) [105]. Additionally, RSV inhibits the expression of pro-inflammatory cytokines, such as TNF-α and IL-1β, by modulating transcription or enzymatic activity in a dose-dependent or context-dependent manner [105,106].

Moreover, RSV enhances AMPK/SIRT1 activation, which inhibits hypoxia-inducible factor 1 alpha (HIF-1α) and vascular endothelial growth factor (VEGF) [107,108]. This effect integrates metabolism regulation with the inflammation pathway via SIRT1 deacetylation of both NF-κB p65 and forkhead box P3 (FOXP3) transcription factors [109]. This activity also leads to Th17 and Tregs regulation in autoimmunization and inflammation diseases [110].

Additionally, the inhibition of HIF-1α and VEGF reveals that RSV reduces inflammation and hypoxia driven by angiogenesis [107,108]. VEGF has also been implicated in physiological angiogenesis and not just pathological angiogenesis, such as in chronic diseases and tumor angiogenesis, and the suppression by RSV has to be examined more deeply [111].

RSV treatment also elevates superoxide dismutase (SOD) levels by increasing the expression of antioxidant enzymes in an SOD-dependent manner and, therefore, alleviates the inflammatory effect of oxidative stress [107,110]. ROS are known to promote further cytokine release, and by alleviating oxidative stress and by boosting the immune regulation loop in cells, RSV acts to decrease the severity and progression of inflammatory diseases.

RSV targets the SIRT1/Nrf2 signaling pathway and therefore decreases NF-κB phosphorylation and acetylation, ROS, and maturation of antigen-presenting cells via SIRT1, leading to reduced chronic inflammation in autoimmune and inflammatory diseases [110].

RSV reduces plasma TNF-α and IL-6 levels in humans but does not alter IL-1 and IL-8 [112]. These results show that a stratified approach should be implemented to test who would benefit more than others with this natural supplement. The lack of effect on IL-1 and IL-8 indicates a potential threshold dose to exert anti-inflammatory effects in a complex milieu like the human plasma.

Also, through miRNA regulation, RSV inhibits activity for both cytokine activity and inflammatory cell production in several inflammatory conditions, indicating that it may function as a systemic anti-inflammatory agent in humans [113].

In autoimmunization and inflammation, RSV inhibits IL-17 expression and, as a result, inhibits activation of Th17 cells in a model of experimental psoriasis [114]. However, as shown, RSV derivatives such as polydatin are not identical to the RSV analog; a careful and comparative pharmacokinetic and bioavailability study would be required to understand the full potential of RSV as a lead-in treatment of inflammatory disorders.

In systemic or liver inflammation, RSV reduces neutrophil infiltration, modulates hepatic transporters, and improves bile acid metabolism [115].

In experimental models with bisphenol A toxicity, RSV reverses metabolic disturbances and restores the expression and activation of glucose metabolism markers to normal levels, reducing oxidative stress and glucose homeostasis regulation [116].

In obesity or metabolic syndrome, it increases the polarization of M2 macrophage in adipose tissue [117]. As a result, it also lowers the levels of nitric oxide and the NF-κB p65 transcription factor. However, as noted earlier, the effects on these inflammatory mediators need to be tested in stratified patient populations.

RSV exhibits anti-inflammatory activity in experimental and clinical studies by lowering several inflammatory markers, such as the biochemical markers C-reactive protein, tumor necrosis factor alpha, and interleukin-6, the oxidative marker malondialdehyde, and by increasing the activities of antioxidant enzymes, such as superoxide dismutase, which points out the overlapping and synergistic mechanisms of this dietary compound for both the inflammatory and oxidative process of several diseases [118]. It also decreases acetic acid-induced pleurisy by inhibiting tissue inflammation, leukocyte infiltration, and oxidative damage [119]. RSV reduces carrageenan-induced synovitis [119,120]. Therefore, RSV, a potential antioxidant and anti-inflammatory agent, has the ability to be useful for treating chronic inflammatory diseases.

## 4. Considerations Related to the Therapeutic Usefulness of RSV

### 4.1. Notes on Dietary Recommendations

The wide distribution of RSV-containing foods has allowed research into the link between dietary patterns and prevention of chronic diseases including obesity and metabolic syndrome, which are inevitably accompanied by low-grade chronic inflammatory state [121,122]. Given that the relative abundance of plant polyphenols can vary remarkably depending on the type of food, it is worth investigating whether the variation in RSV intake is correlated with different human health outcomes [45]. Despite the general indication that RSV positively promotes health outcomes, further evaluation on the differences in bioavailability and impact of diets containing this polyphenol, the influence of lifestyle, food composition, and how these factors impact disease prevention is important [121]. Furthermore, the identification of which nutritional recommendations may improve outcomes by taking into consideration RSV dietary intake is a crucial issue. Thus, to determine which nutritional strategies must be employed, comparative research must be developed with different dietary sources to determine which are the most beneficial in relation to RSV intake [122]. Its positive effects are evident in several contexts such as cardiovascular, neurological, aging, immunity, and cancer-related disease models [123,124,125,126]. RSV acts via various pathways, making further research paramount to elucidate its specific anti-inflammatory mechanisms. Although the reported benefits are well documented, many key questions are still unanswered, such as issues concerning dose–response and the variability across populations. Thus, to clarify the full scope of its therapeutic effects, further research must consider the variables of dose and its combination with other bioactive compounds [127,128,129].

### 4.2. Problems with RSV Bioavailability

Regarding the study on the physiological effects of RSV, it is important to take into account its pharmacokinetic characteristics [130,131,132]. Although it presents good oral absorption (~75%), RSV’s bioavailability is low, less than 1%, mainly due to its rapid metabolism in the intestinal and liver environment into glucuronidated and sulfated metabolites, which results in it having a minimal effect at a systemic level [12,45,124,129]. Due to these features, researchers have been investing in drug-delivery methods in order to increase RSV’s bioavailability, for example, by nanoparticles, liposomes, or by its inclusion into cyclodextrin complexes [66,124,133,134]. This approach allows more effective delivery to the target cells and thus maximizes its positive effects.

### 4.3. Issues Related to the Interpretation of RSV Activity Studies

These bioavailability features present some limitations in transposing in vitro data into in vivo contexts [135]. When assessing a pharmacological agent, the effects must be considered at its physiological dose range. In experimental scenarios, very high RSV doses, which are probably not achievable by dietary intake, have been frequently employed, bringing into question the validity and practicality of these studies in real-world scenarios [135,136,137]. Thus, future in vivo trials must address the discrepancies between in vitro and in vivo results by considering the effects of physiological doses of RSV on human physiology. Besides that, further research on RSV’s glucuronide and sulfate derivatives is needed, since these are the primary circulating forms after its intake, and there are reports that these metabolites retain or even exceed some of the properties of their parent compound [138]. Thus, exploring the effects and possible use of RSV metabolites may offer new insights for future therapeutic interventions [139,140].

Another point for further investigation is the fact that not all the effects found in vitro or in animal models are replicated in clinical studies. Furthermore, the inter-individual response to RSV intake presents a broad range of results; therefore, more investigations are needed to clarify this variability by integrating factors such as the inter-individual metabolic response, dose effects, and environmental variables (genetics, gut microbiota, etc.) that may be responsible for influencing its impact [141,142]. Thus, understanding inter-individual differences will contribute to establishing more effective guidelines for treatment and disease prevention with RSV-based nutraceuticals.

At the cellular and molecular level, RSV has been shown to affect multiple target systems in numerous cell and tissue types, including nuclear and cell-surface receptors, metabolic enzymes, and inflammatory gene signaling pathways [143]. One of the most important anti-inflammatory mechanisms is its inhibition of transcriptional factors like NF-κB and activator protein-1 (AP-1), and key regulators of pro-inflammatory genes, such as COX-2 and iNOS, resulting in the downregulation of these proteins which reduces prostaglandin and nitric oxide (NO) production [123,144,145]. Moreover, RSV modulates epigenetic modulators, most notably SIRT1, affecting gene expression, cellular energy metabolism, inflammation, and immunity [146]. Furthermore, its inhibitory effects on inflammatory responses are also seen through regulation of toll-like receptor (TLR) signaling, which is one of the main initiators of innate immunity [46,144,147]. In this context, its effect on prostaglandin synthesis, by regulating the activity of the COX enzymes, is pivotal in regulating inflammation, both acute and chronic [124,145,148]. In addition, evidence exists that RSV is also effective in the regulation of pro-inflammatory cytokine synthesis, such as TNF-α, IL-1β, and IL-6, also inhibiting IL-8 and monocyte chemoattractant protein-1 (MCP-1) expression by macrophages, which are all mediators that contribute significantly to the aggravation of chronic inflammatory diseases [46,146,149]. RSV also affects various aspects of the immune system, both innate and adaptive immunity, including pro- and anti-inflammatory cytokines, chemokines, and lymphocytes [146]. A significant part of its effect is due to its ability to attenuate the formation of prostaglandin E2 [147]. Furthermore, it promotes a decrease in the production of reactive oxygen species (ROS) involved in inflammation. Regarding its neuroprotective potential, the neuroinflammation process mediated by resident immune cells, microglia and astrocytes, and recruited leucocytes can be modulated by RSV. In vitro and in vivo studies have found evidence that this compound reduces their activation [147]. The aforementioned mechanisms indicate a valuable role in regulating immune–inflammatory processes; therefore, more research must be performed to confirm these anti-inflammatory effects in patients suffering from those diseases.

RSV’s health beneficial impact is likely dependent upon several factors, with dose and exposure time being the most obvious [46]. Furthermore, the health benefits can also depend on the stage of the disease [149]. Therefore, an interesting topic for future investigation is the impact of RSV on the mechanisms that affect intestinal immune function and the regulation of cellular and energy metabolism within immune cells. In addition, due to the fact that RSV is effective in the reduction in cell proliferation and angiogenesis, its role in the management of severe gynecological diseases such as endometriosis needs to be investigated, since chronic inflammation is often implicated in the pathogenesis of the disease by increasing the synthesis of pro-inflammatory prostaglandins by activating COX enzymes [148]. Thus, RSV is a beneficial dietary compound in preventing chronic inflammatory diseases, and consequently in maintaining and promoting better health, thus allowing further investigation as a component in nutraceutical therapies [148]. RSV presents many interesting features, and further studies are needed to characterize the full scope of its anti-inflammatory actions and its impact on the management of human chronic inflammatory and autoimmune diseases.

## 5. Summary and Future Perspectives

Much evidence suggests that RSV plays an encouraging role in the prevention and treatment of many autoimmune, inflammatory, neurological, and neoplastic diseases. It has been shown that RSV modulates many cellular and molecular mediators of inflammation, but its mechanisms of action are complex, including many signaling pathways, and are not completely known yet.

Therefore, future studies should focus on ❶—further evaluation of RSV properties in the course of clinical trials, with improved bioavailability, and ❷—explanation of basic mechanisms of action of RSV in various physiologic states, to make its application the most modern treatment strategy in the prevention and therapy of a broad spectrum of chronic diseases.

## Data Availability

No new data were created or analyzed in this study.

## Abbreviations

AA	arachidonic acid
AMPK	AMP-activated protein kinase
AP-1	activator protein-1
BMK1	big mitogen-activated protein kinase 1, also known as extracellular signal-regulated kinase 5 (ERK5) or mitogen-activated protein kinase 7 (MAPK7)
cAMP	cyclic adenosine monophosphate
cGMP	cyclic guanosine monophosphate
CAT	catalase
c-Jun	a component of the transcription factor AP-1
CNS	central nervous system
Con A	concavalin A
COX-1, COX-2	cyclooxygenases 1 and 2, respectively
cPLA2	cytosolic phospholipase A2
CREB	cAMP response element-binding protein
CYP-450	cytochrome P450
DAMP	damage-associated molecular patterns
DAPK1	death-associated protein kinase 1
ER	endoplasmic reticulum
ERCC1	the excision repair cross-complementation group 1 (protein)
ERK1/2	extracellular signal-regulated kinases 1/2
FDA	U.S. Food and Drug Administration
FOXP3	forkhead box P3 transcription factor
GPX	glutathione peroxidase
H_2_O_2_	hydrogen peroxide
HIF-1α	hypoxia-inducible factor 1 alpha
HO-1	heme oxygenase 1
IκBα kinase and IκBβ kinase	inhibitors of nuclear factor kappa B α and β, respectively
IFN-γ	interferon γ
IKK	IkappaB kinase or IκB kinase
IL-1α, IL-1β, IL-2, IL-6, IL-8, IL-12, IL-17	interleukins: 1-alpha, 1-beta, 2, 6, 8, 12 and 17, respectively
iNOS	inducible nitric oxide synthase
JAK/STAT	Janus kinase-signal transduction and transcription activation
JNK	c-Jun N-terminal kinase
LOX	lipoxygenase
LPS	lipopolysaccharide
MAPK	mitogen-activated protein kinases
MCP-1	monocyte chemoattractant protein-1
MDA	malonyl dialdehyde
MMP-1, MMP-3 and MMP-13	matrix metalloproteinases
mTOR	mechanistic target of rapamycin
MYD88	myeloid differentiation primary response 88, an adaptor protein
NAD^+^	nicotinamide adenine dinucleotide
NADPH	nicotinamide adenine dinucleotide phosphate
NEMO	nuclear factor kappa-light-chain-enhancer of activated B cells (NF-κB) essential modulator
NF-κB	nuclear factor kappa-light-chain-enhancer of activated B cells
NLC	nanostructured lipid carriers
NLRP3 inflammasome	nucleotide-binding oligomerization domain (NOD), leucine-rich repeat (LRR)-containing protein (NLR) family member 3 inflammasome
NO	nitric oxide
NOS	nitric oxide synthase
NOX4	nicotinamide adenine dinucleotide phosphate oxidase type 4
Nrf2	nuclear factor erythroid 2 (NF-E2)-related factor 2
NSAID	non-steroid anti-inflammatory drugs
p38	class of mitogen-activated protein kinases
p65(RelA)	transcription factor p65, also known as nuclear factor NF-κB p65 subunit (RelA proto-oncogene)
PAMP	pathogen-associated molecular patterns
PEPCK	phosphoenolpyruvate carboxykinase
PGE_2_, PGD_2_, PGI_2_	prostaglandins E_2_ D_2_ and I_2_, respectively
PI-3K	phosphoinositide 3-kinase
PIH	pregnancy-induced hypertension
PKC	protein kinase C
PMA	phorbol 12-myristate 13-acetate, also known as 12-O-tetradecanoylphorbol 13-acetate (TPA)
PRR	pattern-recognizing receptors
RAGE	receptors for advanced glycation end products
ROS	reactive oxygen species
RSV	resveratrol
SIRT1	sirtuin 1, an NAD^+^-dependent deacetylase
SLN	solid lipid nanoparticles
SOCS1	suppressor of cytokine signaling 1
SOD	superoxide dismutase TLR—toll-like receptors
TCR	T cell receptor
TLR2, TLR4	toll-like receptor 2 and 4, respectively
TNF-α	tumor necrosis factor alpha
TX	thromboxane
TxA2	thromboxane A2
TPA	12-O-tetradecanoylphorbol 13-acetate, also known as phorbol 12-myristate 13-acetate (PMA)
TRAIL	tumor necrosis factor-related apoptosis-inducing ligand
UVB	ultraviolet B radiation
VEGF	vascular endothelial growth factor
